# Allochthonous zoonotic sporotrichosis in the Brazilian Central-West: diagnostic challenges in a nonendemic area

**DOI:** 10.1590/S1678-9946202567030

**Published:** 2025-05-26

**Authors:** Thaís Badini Vieira, Sirlei Franck Thies, Luana Candido Dias, Brenda Mênick de Oliveira, Flávio Silveira, Juliana Maciel Cassali Vieira, Eriana Serpa Barreto, Angélica Cavalheiro Bertagnolli Rodrigues, Mário de Menezes Coppola, Clairton Marcolongo-Pereira, Renata Osório Faria, Angelita dos Reis Gomes

**Affiliations:** 1 Universidade Federal do Mato Grosso, Campus Sinop, Sinop, Mato Grosso, Brazil; 2 Secretaria de Estado de Saúde de Mato Grosso, Escritório Regional de Saúde de Sinop, Sinop, Mato Grosso, Brazil; 3 Centro Estadual de Diagnóstico e Pesquisa em Saúde Animal Desidério Finamor, Eldorado do Sul, Rio Grande do Sul, Brazil; 4 Centro Universitário do Espírito Santo, Programa de Pós-Graduação em Ciências Veterinárias, Colatina, Espírito Santo, Brazil; 5 Universidade Federal de Pelotas, Faculdade de Veterinária, Departamento de Veterinária Preventiva, Centro de Diagnóstico e Pesquisa em Micologia, Capão do Leão, Rio Grande do Sul, Brazil

**Keywords:** Subcutaneous mycoses, Diagnosis, Zoonosis, One Health, Emerging fungal diseases

## Abstract

Sporotrichosis, a neglected zoonotic fungal infection, is becoming increasingly prevalent in Brazil, with cats being the primary source of human transmission. This report details the first documented case of zoonotic human sporotrichosis in Mato Grosso State, a non-endemic area; the infection was acquired from an animal in an endemic area. The patient developed a subcutaneous ulcerative lesion following contact with a cat from Minas Gerais State, a known disease hotspot. Initially misdiagnosed, the infection was later confirmed as *Sporothrix brasiliensis* after fungal culture and molecular analysis. The patient was successfully treated with itraconazole. This case highlights the importance of considering sporotrichosis in the differential diagnosis, even in non-endemic areas, due to the risk of zoonotic transmission. It also emphasizes the need for a One Health approach to improve surveillance, diagnostic accuracy, and management of emerging fungal diseases in endemic and expanding areas.

## INTRODUCTION

Sporotrichosis is a neglected zoonotic disease with an expanding distribution that exceeds epidemiological estimates. In Brazil, cats are the main hosts and sources of transmission, driving outbreaks across multiple states. Previously considered predominantly sapronotic, transmission occurs mainly via feline scratches and bites^1^. Biofilm formation by *Sporothrix* species, especially in feline claws, has been implicated in the zoonotic transmission^2^.

In humans, sporotrichosis usually manifests in a fixed cutaneous or lymphocutaneous form. Fixed cutaneous form presents as localized papules or ulcers at the inoculation site. In contrast, the lymphocutaneous form is characterized by ascending nodular lesions along lymphatic pathways, which may ulcerate, fistulize, and discharge purulent material^3^.

Disseminated sporotrichosis is more common in immunocompromised patients, particularly people living with HIV (PLWHIV), and can affect multiple organs, mimicking other opportunistic infections. Its diverse clinical presentation, including ulcerated nodules and mucosal or molluscum-like lesions, hinders diagnosis and requires its inclusion in the differential diagnosis of cutaneous lesions^4^, even in non-endemic areas. This report documents the first recorded case of zoonotic sporotrichosis in Mato Grosso State, with a confirmed origin in another state. The patient acquired the infection in an endemic area of Minas Gerais State, but was diagnosed and treated in Mato Grosso State, a non-endemic area. This geographic disconnect likely contributed to the delayed diagnosis.

## CASE REPORT

**Figure 1:**
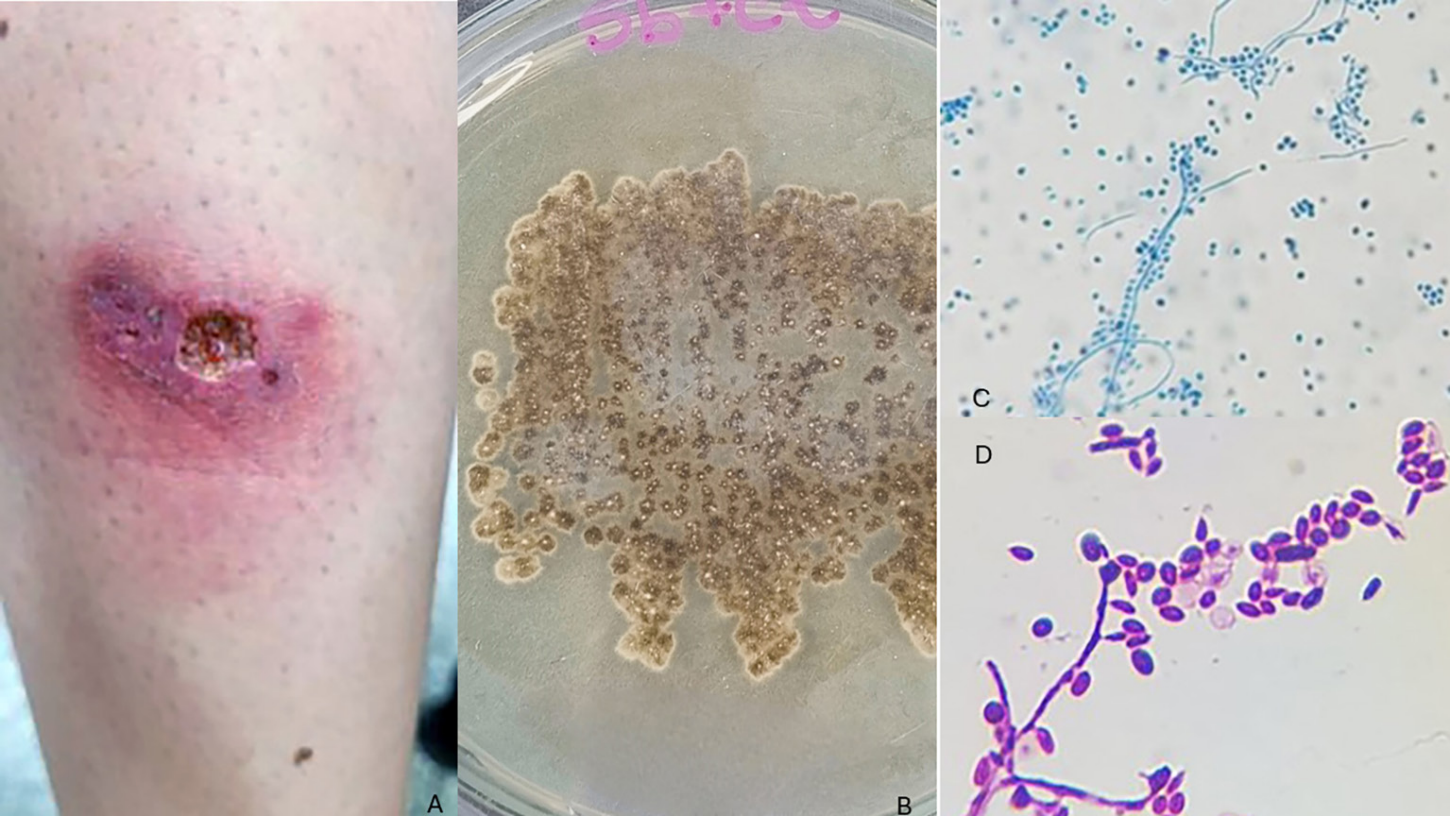
Morphological and microscopic characteristics of *Sporothrix brasiliensis*: (A) Ulcerative lesion on the leg caused by *Sporothrix brasiliensis*, characterized by well-defined erythematous borders and a necrotic center, indicative of the fixed cutaneous form of sporotrichosis; (B) *Sporothrix brasiliensis* colony on Sabouraud agar at 25 °C after 12 days of incubation, showing filamentous growth with a rough texture, velvety surface, and dark brown pigmentation; (C) *Sporothrix brasiliensis* visualized with lactophenol cotton blue staining at 40x magnification, displaying slender septate hyphae and pyriform conidia arranged in rosette-like clusters, typical of the filamentous phase; (D) *Sporothrix brasiliensis* visualized with Gram staining at 100x magnification, showing elliptical yeast cells in the yeast phase.

A 23-year-old female university student residing in Sinop city, Mato Grosso State, Brazil (11° 52’ 23" South, 55° 29’ 54" West), presented with a nodule on her left leg, which was first noticed on February 15, 2023. By March 16, the lesion had enlarged and ulcerated, prompting her to seek medical attention at the Emergency Care Unit in Sinop city. Clinical examination revealed a fixed, exudative, ulcerated lesion accompanied by pain and pruritus (Figure 1A).

The patient, originally from Esmeraldas city, Minas Gerais, had recently traveled home for the holidays to visit her family. Esmeraldas city is an endemic area for animal sporotrichosis, with a high incidence of feline cases. Approximately four weeks before the lesion appeared, she had contact with her family's cat, which had been diagnosed with sporotrichosis based solely on clinical signs and was undergoing antifungal treatment. No respiratory symptoms were observed in the cat. Despite ongoing treatment, the cat still had active ulcerated lesions, particularly on its paws, which had not fully healed.

The patient had no history of gardening or other potential traumas that could have led to sapronotic transmission. She reported frequent handling of cats, but did not recall being scratched or bitten. Since no lesions were visible on her skin and she did not experience any direct injury from the animal, she did not initially suspect sporotrichosis, as she was unaware of the zoonotic transmission risk associated with contact with cats.

A swab sample from the lesion was subjected to cytological examination, revealing oval or round yeast cells with a whitish halo under rapid panoptic staining. Another sample was sent to the Central Laboratory of Mato Grosso State for fungal culture on mycobiotic agar (Mycosel^®^) and was incubated at 25 °C for 20 days. The colonies grew slowly, displaying a moist, velvety texture with dark brown to black pigmentation (Figure 1B). Microscopic analysis using cotton-blue lactophenol staining revealed hyphae and conidiophores with daisy-like conidia, consistent with *Sporothrix* spp. (Figures 1C and 1D).

The isolates were sent to the Center for Diagnostic and Research in Mycology at the Federal University of Pelotas for confirmation. The colonies were subcultured on Sabouraud dextrose agar (37 °C) and mycobiotic agar (25 °C) to assess dimorphism. DNA was extracted using a standard Tris-HCl buffer protocol, followed by mechanical disruption with glass beads. PCR targeting the ITS region of ribosomal DNA (primers ITS1/ITS4) and calmodulin (CAL) gene amplification (primers CL1/CL2) was performed for species identification^5^. Sequencing confirmed the isolate as *Sporothrix brasiliensis*.

The patient was treated with oral itraconazole (100 mg daily) for three months, leading to complete clinical resolution of the lesion. This study was approved by the Research Ethics Committee of the Centro Universitario do Espirito Santo (CEP/UNESC), under protocol N° 7.012.264 (CAAE: 82391524.5.0000.5062). Patient confidentiality and privacy were strictly preserved, and all personal identifiers were removed. Written informed consent was obtained from the patient for publication of her medical information. This study adhered to the ethical guidelines established by the Brazilian National Health Council (Resolution N° 466/2012).

## DISCUSSION

This case highlights the importance of identifying sporotrichosis in non-endemic areas, such as the Mato Grosso State. Zoonotic sporotrichosis in Brazil has evolved from isolated cases to a growing endemic disease. First reported in Sao Paulo State^6^, it became endemic in Rio de Janeiro and Rio Grande do Sul States in the 1990s, later spreading to Minas Gerais State and more recently to Parana, Rio Grande do Norte, and Goias states^7,8^, reinforcing the need for increased surveillance.

The expansion of sporotrichosis reflects gaps in epidemiological surveillance, with silent zones facilitating hidden transmissions. Urbanization and dispersal corridors are critical factors, underscoring the urgency of mandatory reporting and strategic monitoring to contain its spread^8^.

Although the patient did not recall any visible injuries, the lack of evident trauma did not rule out the possibility of *Sporothrix brasiliensis* infection. Transmission may occur via microlesions or unnoticed skin abrasions^9^, potentially due to contact with exudates from ulcerated lesions. Therefore, sporotrichosis should be considered in the differential diagnosis, even in the absence of a clear trauma history related to contact with cats. While the animal was undergoing antifungal treatment, it reduced the fungal load^10^, but did not eliminate the risk of transmission, particularly when active lesions persisted. Thus, although *Sporothrix brasiliensis* has also been found to have a significant presence in environmental sources, suggesting its ability to survive and thrive in diverse ecological niches^11^, sapronotic transmission remains unlikely.

The One Health approach emphasizes the interconnection between human, animal, and environmental health, which is essential to manage zoonotic diseases^8^. Although the Brazilian Ministry of Health previously included sporotrichosis in its Health Surveillance Guide without making it nationally notifiable^12^, human sporotrichosis will be added to the National List of Compulsory Notification and must be registered in the Notifiable Diseases Information System (SINAN) starting January 30, 2025. This decision was officially formalized during the 1^st^ Meeting of the Tripartite Interagency Commission (CIT) in 2025, in accordance with Brazil's existing regulation for compulsory disease notification.

According to the Brazilian Ministry of Health, compulsory notification is required for diseases that pose public health risks, holds outbreak potential, or exhibit significant epidemiological changes^13^. The inclusion of human sporotrichosis reflects the recognition of these factors, particularly given the zoonotic nature of the disease and its increasing prevalence in certain regions^14–16^. However, the unpredictability of fungal diseases, exacerbated by climate change, continues to complicate control efforts^17^. Early diagnosis and timely intervention could prevent up to half of fungal-related deaths^18^, underscoring the importance of robust surveillance and notification systems to mitigate the impact of this and other emerging diseases.

The patient was treated with itraconazole 100 mg/day for 12 weeks, resulting in clinical resolution. However, Brazilian guidelines recommend itraconazole 200 mg/day for three to six months for cutaneous and lymphocutaneous forms^3^. In severe or disseminated cases, liposomal amphotericin B (three to five mg/kg/day) is indicated, followed by itraconazole (200 mg/day) for at least 12 months^5^. These differences highlight the need for individualized treatment strategies based on disease severity and patient response.

## CONCLUSIONS

This report documents the first confirmed case of zoonotic sporotrichosis diagnosed and treated in Mato Grosso State, Brazil, with the infection acquired in Minas Gerais State, an endemic region. This case underscores diagnostic challenges faced by non-endemic regions, highlighting the importance of considering zoonotic sporotrichosis in the differential diagnosis, even in areas where the disease is not traditionally recognized. Additionally, it emphasizes the need for a One Health approach that integrates human, animal, and environmental health to address emerging zoonotic diseases and improve surveillance and diagnosis in endemic and non-endemic regions.
